# Supplementary material for: Patterns of Intron Gain and Loss in Fungi
Source: PLoS Biol. 2004 Nov 30;2(12):e422. doi: 10.1371/journal.pbio.0020422 (PMC532390; doi:10.1371/journal.pbio.0020422)
Supplement: Table S1 — Also available at http://genes.mit.edu/NielsenEtAl/. (4.3 MB ZIP). [file pbio.0020422.st001.zip › NielsenEtAl/html/393.html]

AN5127.1.NCU01741.1.MG07189.1.FG01435.1


```
 CLUSTAL W (1.82) Multiple Sequence Alignments - Introns Inserted


Sequence 1: MG07189.1	121 aa
Sequence 2: FG01435.1	174 aa
Sequence 3: NCU01741.1	180 aa
Sequence 4: AN5127.1	168 aa
Alignment Length: 186 aa
Number Identitical Residues: 46 aa
Alignment Score (without introns) 2808


MG07189.1 	MASS2R~FGPSSLHQRDPR--SALFEGYNG-GADSNRRPSPSPNR--AAGGYGYPGGP--
NCU01741.1	-MAS~R2FAHSSLHQRDPR--SDLFKGYSGSSAAERRSANASPARYGGGGGYGYGGGYGG
FG01435.1 	-MAQ~R2FGASSLHQRDSR--SALFEGYNG-DAASRRPVSASPNRGYGYGGYGAPSPS--
AN5127.1  	-MAS~R2FSRSDLHRRDPRASSSLFDSYDG----ISRPASKSPGN---VGGYGYGSYP--
          	  :. * *. *.**:**.*::* **..*.*      *  . ** .    ****  .    

MG07189.1 	------ASNHLGVQGS---SYRPATPNKR2GQYSDAVLNELESQNDSQVEGIMGKVRILK
NCU01741.1	GGSYNGSSSTLGMERG---GFRPATPNSR~GQYSDAVLNELESQNDEQVEGIMGKVKILK
FG01435.1 	------PLGHGGFDSSRPAPFRSATPNKR~GQYSDAVLNELESQNDAQVEGILGKVKVLK
AN5127.1  	-------GADRSMNGNGYTGYKSAMPNSK2GHYSDAVLSHLESQNEEEVEGITAKVRMLK
          	           ..: .  : ::.* **.: *:******..*****: :**** .**::**

MG07189.1 	D0L--------RHNSTTTARY--------------------YEQDVGDG-----------
NCU01741.1	D0MTIAIGDEIRDSSALAEKMNESFDQTRLRLRGTMNRMLIMAQKTNVGWRVWLAFFAAV
FG01435.1 	D0MTVAIGDEIRESSALAEKMNDTFDSTRLRLRGTMNRMLVMAQRTGVGWKVWLIFFAAV
AN5127.1  	D0LTLAIGDEIRDTTHISDLN-DSFENTRTRIRGNMNRMMRMAERTGVGWKVWAGFFALV
          	* :: : ... *..:  :    .: ..:     .. .      : .. *        :  

MG07189.1 	----------
NCU01741.1	VFLFFYVRYL
FG01435.1 	IMLFMYVWLF
AN5127.1  	FFLFFYVWIT
          	
```
